# Genetic testing for Prader-Willi syndrome and Angelman syndrome in the clinical practice of Guangdong Province, China

**DOI:** 10.1186/s13039-019-0420-x

**Published:** 2019-02-18

**Authors:** Chang Liu, Xiangzhong Zhang, Jicheng Wang, Yan Zhang, Anshi Wang, Jian Lu, Yanlin Huang, Shu Liu, Jing Wu, Li Du, Jie Yang, Hongke Ding, Ling Liu, Xin Zhao, Aihua Yin

**Affiliations:** 1 Medical Genetic Center, Guangdong Women and Children Hospital, Guangzhou, Guangdong 510010 Peoples, Republic of China; 2 Maternal and Children Metabolic-Genetic Key Laboratory, Guangdong Women and Children Hospital, Guangzhou, 510010 Guangdong China; 3 Children Inherited Metabolism and Endocrine Department, Guangdong Women and Children Hospital, Guangzhou, 510010 Guangdong China; 4 Department of Neonatology, Guangdong Women and Children Hospital, Guangzhou, 510010 Guangdong China; 5 Department of Hematology, The Third Affiliated Hospital of Sun Yat-sen University, Guangzhou, 510630 Guangdong China

**Keywords:** Prader-Willi syndrome, Angelman syndrome, Genetic testing, Clinical practice

## Abstract

**Background:**

Prader-Willi syndrome (PWS) and Angelman syndrome (AS) are clinically distinct neurodevelopmental disorders caused by absence of paternally or maternally expressed imprinted genes on chromosome 15q11.2-q13.3 region.

**Methods:**

3331 individuals was recruited from June 2013 to December 2016 under an institutional review board-approved protocol of informed consent. The methylation-specific PCR was employed as a first-tier screening test. The multiplex-fluorescent-labeled STR linkage analysis was carried out to define the underlying genetic mechanisms. The chromosomal microarray analysis was employed to identify chromosomal breakpoints in confirmed cases, and to detect other chromosomal abnormalities in undiagnosed cases. Genetic counseling and recurrence risk assessment were provided to families with affected individuals.

**Results:**

The methylation-specific PCR identified 36 PWS suspected patients and 13 AS suspected patients. *UBE3A* sequence analysis identified another 1 patient with AS. The STR linkage analysis define the underlying genetic mechanisms. Thirty PWS patients were with paternal deletions on chromosome region 15q11-q13, 5 with isodisomic uniparental disomy and 1 with mixed segmental isodisomic/ heterodisomic uniparental disomy of maternal chromosome 15. Twelve AS patients were with maternal deletions, 1 with isodisomic uniparental disomy and 1 with *UBE3A* gene mutation. The chromosomal microarray analysis identified chromosomal breakpoints in confirmed cases, and detected chromosomal abnormalities in another 4 patients with clinically overlapped features but tested negative for PWS/AS. Genetic counseling was offered to all families with affected individuals.

**Conclusions:**

Identifying the disorders at early age, establishing the molecular mechanisms, carrying out treatment intervention and close monitoring can significantly improve the prognosis of PWS/AS patients.

## Background

Prader-Willi syndrome (PWS, OMIM ref. 176,270) and Angelman syndrome (AS, OMIM ref. 105,830) are clinically distinct neurogenetic disorders with multiple phenotypic manifestations. PWS patients present with neonatal hypotonia, poor sucking and weak cry in the postnatal period, delayed psychomotor development and hyperphagia in early childhood, severe obesity, short stature and hypogonadism in adolescents [1]; The initial symptoms of AS overlap with a lot of other disorders, and more characteristic features present later in childhood, including microcephaly, severe developmental delay, gait ataxia and/or tremulousness of the limbs, seizures, absent or severely limited speech, and a unique behavior with happy demeanor [2]. Each syndrome occurs with an estimated prevalence of 1:15,000–1:25,000 live births [3].

The two clinically distinct disorders are both caused by genetic alterations in chromosome region 15q11.2–q13. PWS is attributed to deficiencies of paternally expressed genes, usually as the consequence of paternal deletion (65–70%), maternal uniparental disomy (UPD(15)mat, 20–30%), mutations/ deletions of the imprinting centre (2–5%) and a translocation of the imprinting center (< 1%) [2, 3]. AS arises from molecular defects including maternal deletion (70–75%), paternal uniparental disomy (UPD(15)pat, 2–7%), *UBE3A* gene mutations (5–10%) and mutations/ deletions of the imprinting centre (3–5%) [3, 4]. Establishing the molecular mechanism will provide information on possible clinical features, prognosis and recurrence risk [5].

## Methods

### Ethics statement

The study has been approved by Medical Ethics Committee of Guangdong Women and Children Hospital. Written informed consent was obtained from all participants, their parents or legal guardians (in the case of children under 16). Authors had access to information that could identify individual participants, and the information was anonymized prior to submission. All the procedures performed in the study were in accordance with the Declaration of Helsinki.

### Patients and samples

Key Laboratory of Inherited Metabolic Diseases under Health Department of Guangdong Province was set up in Guangdong Women and Children Hospital, and has been served as a tertiary referral centre for the province. The centre provides comprehensive genetic counseling, diagnostic and laboratory service for suspected patients and their families. 290 patients suspected for PWS/AS were referred for further diagnosis and genetic confirmation, and 3041 neonates with initial symptoms of PWS/AS were recommended for the genetic screening. The study cohort of 3331 individuals was recruited from June 2013 to December 2016 under an institutional review board-approved protocol of informed consent.

### Clinical manifestations

Of the 3331 suspected individuals, 3241 were neonates (≤ 28 days) presented with hypotonia, poor responsiveness, feeding difficulty and weak cry; 49 were infants (1 month to 1 year of age) demonstrated with hypotonia and delayed psychomotor development; another 39 children (1 year to 15 years of age) were referred to the center because of some further characteristic behaviors of PWS or AS. Given that both PWS and AS have clinical overlaps with other diseases, it is difficult to diagnose them solely based on clinical manifestations.

### Genetic diagnosis

Blood samples were obtained from all participants and genomic DNA was isolated from the whole blood by standard procedures using the DNA isolation mini kit (ZEESAN, Xiamen, China) and Lab-Aid 820 nucleic acid extraction instrument (ZEESAN, Xiamen, China).

#### Methylation-specific PCR analysis

Genomic DNA was treated with sulfite using the CpGenome Turbo Bisulfite Modification Kit (Millipore, CA, USA) according to the manufacturer’s instructions. The imprinting gene SNRPN containing a potential imprinting center for a chromosome domain on chr15q11–13. Almost all CpG dinucleotides are methylated on the maternal chromosome and unmethylated on the paternal chromosome. The methylated SNRPN locus was amplified with the MF 5’-TAAATAAGTACGTTTGCGCGGTC-3′ and MR 5’-AACCTTACCCGCTCCATCGCG-3′ to generate the 174 bp methylation product. While the non-methylation primers PF 5′-GTAGGTTGGTGTGTATGTTTAGGT-3′ and PR 5’-ACATCAAACATCTCCAACAACCA-3′, were used to amplify 100 bp of the non-methylated allele.

#### STR linkage analysis

The multiplex-fluorescent-labeled STR linkage analysis was performed as previously described^.^ [2] The microsatellite markers were selected according to their high heterozygosity and locations in the typical deletion region and adjacent regions. Seven STR markers D15S11, D15S646, D15S817, D15S128, D15S1513, GABRB3, D15S822 located in the typical PWS/AS deletion region of BP1 to BP3, and two loci D15S659, FES in the distal region near telomere. The experimental procedures were as previously described^.^ [2] GeneMapper software (Applied Biosystems) was used for data collection and allele sizing.

#### Chromosomal microarray analysis

The chromosomal microarray analysis (CMA) was carried out to identify chromosomal breakpoints in confirmed cases. And in undetected cases, it was employed to detect other chromosomal abnormalities. From 2012 to 2014, Agilent’s 8 × 60 K commercial array (Agilent Technologies, CA, USA) was employed for array-based CGH analysis, and Agilent Genomic Workbench Lite Edition 6.5.0.18 software (Agilent Technologies) was used for data analysis. From 2015 onwards, CMA was performed with CytoScan 750 K array (Affymetrix, CA, USA), and Affymetrix Chromosome Analysis Suite software was used for genotype calling, quality control and CNV identification.

## Results

### Methylation-specific PCR analysis

**Fig. 1:**
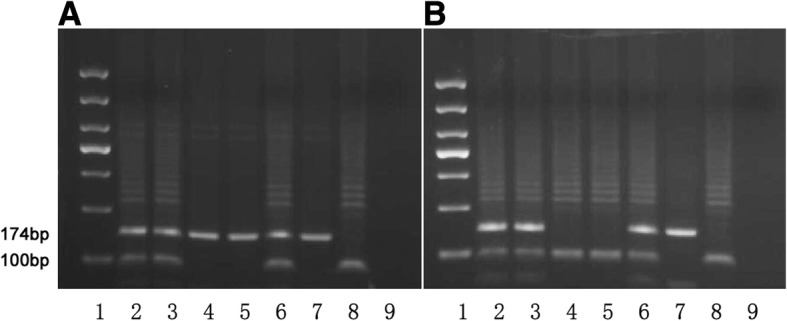
MS-PCR analysis of SNRPN gene in PWS/ AS patients. MS-PCR products of 174 bp and 100 bp are amplified from methylated and unmethylated alleles of the SNRPN gene locus respectively. **a** Lane 1, 1000-bp DNA ladder marker; Lane 2, patient’s father; Lane 3, patient’s mother; Lane 4 and 5, PWS patient from case 2; Lane 6, negative control; Lane 7, PWS positive control; Lane 8, AS positive control; Lane 9, blank control. **b** Lane 1, 1000-bp DNA ladder marker; Lane 2, patient’s father; Lane 3, patient’s mother; Lane 4 and 5, AS patient from case 38; Lane 6, negative control; Lane 7, PWS positive control; Lane 8, AS positive control; Lane 9, blank control

The PCR products of 174 bp and 100 bp were obtained from methylated and unmethylated alleles of *SNRPN* gene locus. In 13 patients, the MS-PCR results demonstrated absence of maternal allele at 15q11-q13 or deletion of a methylated CpG island at the *SNRPN* gene (presented with only 100 bp of paternal fragment), were suspected for AS; 36 patients’ analysis results showed the paternal chromosome is aberrantly methylated at this region (presented with only 174 bp of maternal fragment), and were suspected for PWS (Fig. 1).

The MS-PCR results of another 3281 participants presented normal (with both 100 bp and 174 bp fragments). Clinical re-assessments were carried out. For those individuals presented with characteristic features of AS, *UBE3A* sequence analysis were recommended; For those presented with mild or atypical symptoms, further examinations and adequate follow-up were provided; For those demonstrated with severe manifestations, a careful review of the patients’ histories, clinical features and EEG findings were suggested for differential diagnoses.

### STR linkage analysis

**Fig. 2:**
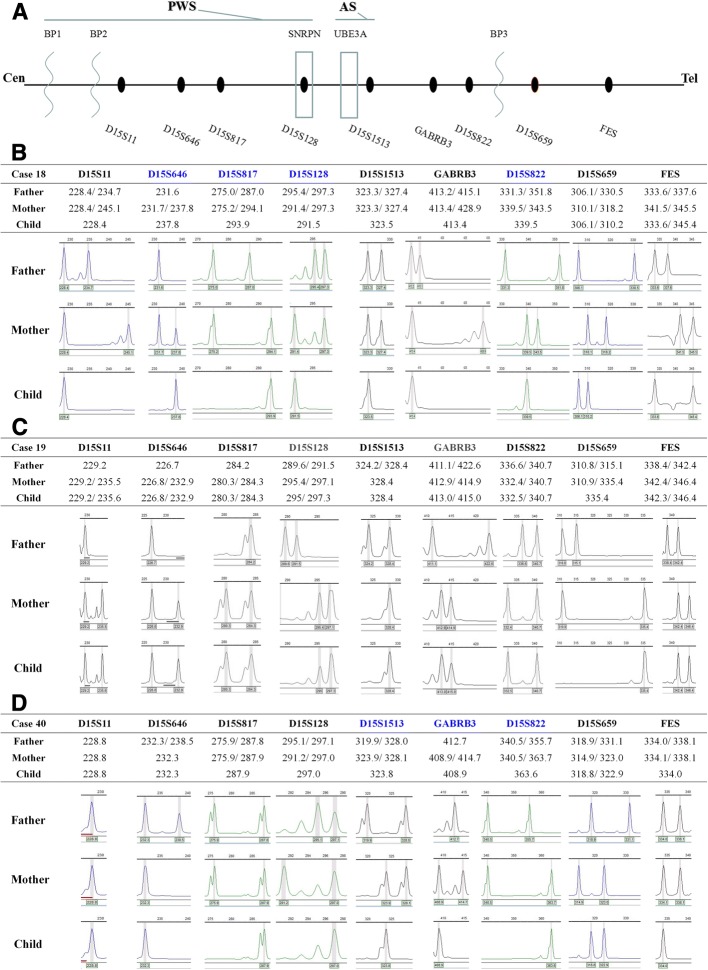
STR linkage analysis results of PWS (deletion type and UPD type) and AS (deletion type). **a** The distribution of nine STR locus. Seven STR loci alleles are located in the typical PWS/AS deletion region of BP1 to BP3, and the remaining two are located in the distal region near the telomere. Cen: centromere; Tel: telomere; BP: breakpoint. **b** Linkage analysis of Case 18 showing the PWS patient has the paternal deletion mutation of chromosome 15 fragment (STR D15S128, D15S646, D15S817, D15S822). **c** Linkage analysis of Case 19 showing the PWS patient carries mixed segmental isoUPD and heteroUPD of maternal chromosome 15 fragment (STR D15S128, GABRB3, D15S659). **d** Linkage analysis of Case 40 showing the AS patient has the maternal deletion mutation of chromosome 15 fragment (STR D15S1513, GABRB3, D15S822)

The multiplex-fluorescent-labeled STR linkage analysis was carried out to define the underlying genetic mechanism in 49 suspected patients and their parents. 13 patients showed the maternal region deletion at the chromosome 15q11-q13 and were confirmed as AS. Of them, one patient carried two chromosome 15 alleles inherited from one paternal chromosome 15, and was identified as AS with isodisomic uniparental disomy type. Moreover, 36 patients demonstrated with the paternal deletion in this region and were diagnosed as PWS. Of them, 5 patients carried two chromosome 15 alleles inherited from one maternal chromosome 15, and was identified as PWS with isodisomic uniparental disomy type. Another one patient was diagnosed with PWS caused by mixed segmental isodisomic/heterodisomic uniparental disomy of maternal chromosome 15. The results of all the patients tested by STR linkage analysis were listed in Table 1, and some representative cases were shown in Fig. 2.

**Table 1:** Demographics and major clinical features

Case No.	Clinical Diagnosis	Sex	Age	Molecular Cytogenetic Test Results	Major Clinical Features
1	PWS	Female	6 h	^a^ del(15)(q?11q?13)pat	Prenatal hypotonia, low prenatal weight and below-average height
2	PWS	Male	10 h	del(15)(q11.2q13.1)pat	Hypotonia in neonate, hypoxic ischemic encephalopathy
3	PWS	Male	11 h	^a^ del(15)(q?11q?13)pat	Poor suck, less spontaneous arousal, and weak cry
4	PWS	Male	12 h	^a^ del(15)(q?11q?13)pat	Poor suck, weak cry, and typical facial features
5	PWS	Male	12 h	^a^ del(15)(q?11q?13)pat	Prenatal hypotonia, atypical fetal position during delivery
6	PWS	Female	13 h	^a^ del(15)(q?11q?13)pat	Poor suck, less spontaneous arousal, and weak cry
7	PWS	Female	14 h	isoUPD(15)mat	Hypotonia in neonate, hypoxic ischemic encephalopathy, and typical facial features
8	PWS	Female	15 h	^a^ del(15)(q?11q?13)pat	Prenatal hypotonia, low prenatal weight and below-average height
9	PWS	Male	16 h	del(15)(q11.2q13.1)pat	Prenatal hypotonia, atypical fetal position during delivery
10	PWS	Male	1 day	^a^ del(15)(q?11q?13)pat	Hypotonia in neonate, hypoxic ischemic encephalopathy, and typical facial features
11	PWS	Male	1 day	^a^ del(15)(q?11q?13)pat	Poor suck, weak cry, and typical facial features
12	PWS	Male	1 day	isoUPD(15)mat	Prenatal hypotonia, low prenatal weight and below-average height
13	PWS	Male	2 days	del(15)(q11.2q13.1)pat	Poor suck, weak cry, and typical facial features
14	PWS	Male	3 days	del(15)(q11.2q13.1)pat	Poor suck, less spontaneous arousal, and weak cry
15	PWS	Male	4 days	del(15)(q11.2q13.1)pat	Lethargy and poor suck
16	PWS	Male	5 days	^a^ del(15)(q?11q?13)pat	Hypotonia in neonate and hypoxic ischemic encephalopathy
17	PWS	Male	6 days	^a^ del(15)(q?11q?13)pat	Poor suck, weak cry, and typical facial features
18	PWS	Male	7 days	^a^ del(15)(q?11q?13)pat	Poor suck, less spontaneous arousal, weak cry, and typical facial features
19	PWS	Female	7 days	segmental iso/heteroUPD(15)mat	Lethargy and poor suck
20	PWS	Female	10 days	^a^ del(15)(q?11q?13)pat	Poor suck, less spontaneous arousal, and weak cry
21	PWS	Male	10 days	^a^ del(15)(q?11q?13)pat	Lethargy and poor suck
22	PWS	Female	11 days	^a^ del(15)(q?11q?13)pat	Lethargy, poor suck, and typical facial features
23	PWS	Female	16 days	isoUPD(15)mat	Thyroid axis dysfunction, poor suck, and weak cry
24	PWS	Female	17 days	^a^ del(15)(q?11q?13)pat	Hypotonia in neonate, hypoxic ischemic encephalopathy
25	PWS	Male	20 days	del(15)(q11.2q13.1)pat	Lethargy and poor suck
26	PWS	Male	24 days	isoUPD(15)mat	Poor suck, less spontaneous arousal, and weak cry
27	PWS	Male	24 days	^a^ del(15)(q?11q?13)pat	Lethargy and poor suck
28	PWS	Male	25 days	del(15)(q11.2q13.1)pat	Lethargy, poor suck, and typical facial features
29	PWS	Male	1 month	^a^ del(15)(q?11q?13)pat	Hypotonia in neonate and respiratory impairment
30	PWS	Male	1 month	^a^ del(15)(q?11q?13)pat	Lethargy, poor suck, and typical facial features
31	PWS	Female	1 month	isoUPD(15)mat	Thyroid axis dysfunction and typical facial features
32	PWS	Female	2 months	^a^ del(15)(q?11q?13)pat	Delayed motor development and typical facial features
33	PWS	Male	3 months	^a^ del(15)(q?11q?13)pat	Delayed motor development and lethargy
34	PWS	Female	1 year	^a^ del(15)(q?11q?13)pat	Delayed motor development and lethargy
35	PWS	Female	4 years	^a^ del(15)(q?11q?13)pat	Obesity, severe intellectual disability, typical facial features
36	PWS	Male	6 years	^a^ del(15)(q?11q?13)pat	Obesity, mental and developmental retardation, short stature, hypogonadism, typical facial features
37	AS	Female	12 h	del(15)(q11.2q13.1)mat	Hypotonia, sucking and swallowing diffculties
38	AS	Male	15 days	del(15)(q11.2q13.1)mat	Hypotonia, sucking and swallowing diffculties
39	AS	Male	15 days	isoUPD(15)pat	Hypotonia, sucking and swallowing diffculties
40	AS	Male	27 days	del(15)(q11.2q13.1)mat	Seizures, delayed psychomotor development
41	AS	Female	1 year	^a^ del(15)(q?11q?13)mat	Seizures, happy disposition, speech defect, intellectual disability
42	AS	Female	1 year	del(15)(q11.2q13.1)mat	Seizures, happy disposition, speech defect, intellectual disability
43	AS	Male	1 year	^a^ del(15)(q?11q?13)mat	Seizures, happy disposition, speech defect, severe intellectual disability
44	AS	Male	2 years	del(15)(q11.2q13.1)mat	Seizures, happy disposition, speech defect, intellectual disability
45	AS	Male	2 years	^a^ del(15)(q?11q?13)mat	Seizures, happy disposition, speech defect, intellectual disability, characteristic EEG pattern
46	AS	Male	2 years	del(15)(q11.2q13.1)mat	Seizures, happy disposition, speech defect, intellectual disability, characteristic EEG pattern
47	AS	Male	2 years	UBE3A mutation	Seizures, happy disposition, speech defect, intellectual disability, characteristic EEG pattern
48	AS	Male	2.9 years	del(15)(q11.2q13.1)mat	Seizures, happy disposition, speech defect, intellectual disability
49	AS	Male	2.4 years	^a^ del(15)(q?11q?13)mat	Seizures, happy disposition, speech defect, intellectual disability, characteristic EEG pattern
50	AS	Female	4 years	del(15)(q11.2q13.1)mat	Seizures, happy disposition, speech defect, intellectual disability, characteristic EEG pattern

The “^a^"indicated that the accurate deletion region on the chromosome was uncertain because of limited numbers of the microsatellite loci

In addition, one AS patient was confirmed by further analysis of *UBE3A* gene. Table 2 shows the pattern of underlying genetic mechanisms in our cohort.

**Table 2:** The pattern of underlying genetic mechanisms in our cohort

Total patients (50)	Microdeletion	UPD	IC defect	UBE3A	Unknown
PWS patients (36)	30	6	0	–	0
Percentage	83.3%	16.7%	0%	–	0%
AS patients (14)	12	1	0	1	0
Percentage	85.7%	7.1%	0%	7.1%	0%

### Chromosomal microarray analysis

**Fig. 3:**
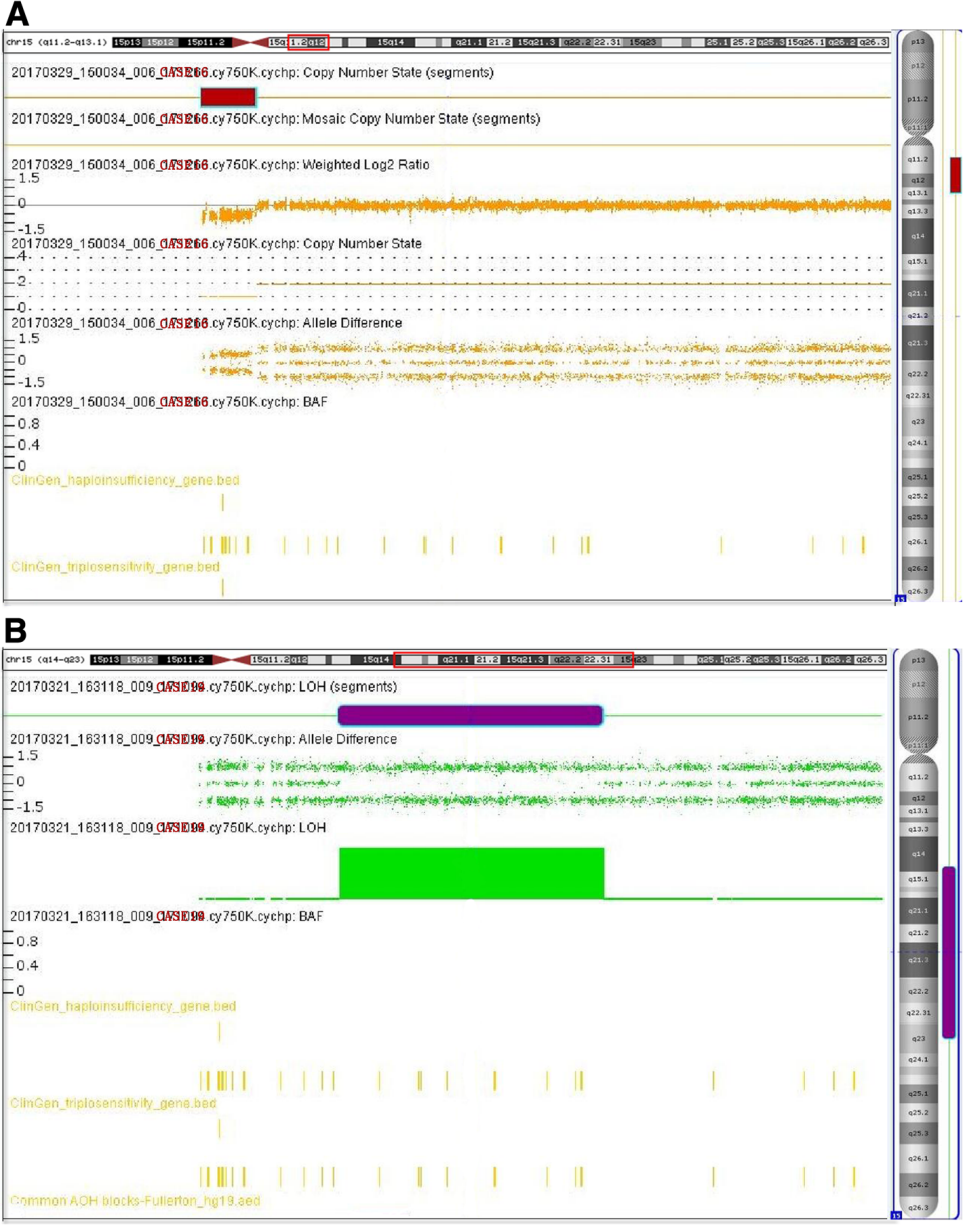
CMA results of some representative cases with PWS/AS**. a** Chromosomal microarray analysis of Case 16 showing the patient has a deletion mutation of chromosome 15. **b** Chromosomal microarray analysis of Case 19 showing the patient has segmental iso/heteroUPD on chromosome 15

For 21 PWS/AS patients, CMA was carried out to validate previous results and to detect the chromosomal breakpoints. The results were in accordance with previous tests and demonstrated with various deletion fragments. The results of some representative cases were shown in Fig. 3. For another 56 subjects presented with severe clinical manifestations but tested negative for PWS/AS, CMA was employed to detect other chromosomal abnormalities. It identified 14 CNVs in 11 out of 56 (19.6%) patients. Among these CNVs, 5 were classified as pathogenic, 6 as VOUS, and 3 as benign. Two pathogenic CNVs were reported in one patient. Therefore, CMA approach identified another 4 patients in 56 individuals with clinically overlapped features but tested negative for PWS/AS. The causative CNVs and their related syndromes were listed in Table 3. The 1p36 microdeletion syndrome resembles PWS presenting with developmental delays/ intellectual disability, craniofacial dysmorphism, hypotonia and other congenital anomalies [3]. The Mowat–Wilson syndrome is caused by heterozygous mutations in ZEB2 gene on chromosome 2, is associated with severe mental retardation, microcephaly, seizures, short stature, and characteristic facial features that resemble those of AS [3]. The 22q13.3 deletion syndrome may present with nondysmorphic facial features, absent or minimal speech, and moderate to severe developmental delay, sometimes with behavioral features in the autism spectrum, and may present with an AS-like phenotype [3]. Another patient with an AS-like condition carried 17p13.2-pter deletion and 22q13.33-qter duplication, presented with brain abnormalities, developmental delay, facial dysmorphisms, hypotonia and seizures.

**Table 3:** The causative CNVs other than 15q11–13 detected by CMA

Causative CNVs	Related Syndrome
arr[hg19] 1p36.33p36.32 (849,466-4,273,842)×1	1p36 microdeletion syndrome
arr[hg19] 2q22.2q22.3 (143,208,691-147,030,229)× 1	Mowat-Wilson syndrome
arr[hg19] 22q13.33 (49,542,105-51,197,766)×1	22q13.3 microdeletion syndrome
arr[hg19] 17p13.3p13.2 (525–5,387,515)×1; 22q13.33 (50,522,375-51,197,766)×3	Miller-Dieker syndrome

## Discussion

The strategies for the analysis of PWS/AS can be affected by many factors, including the arrangement of laboratory services, the coverage of medical insurance and the patterns of referral. [6] In the present study, the MS-PCR approach was employed as a first-tier screening test to detect abnormal parent-specific methylation within the PWS and AS critical region. It is a rapid and convenient platform, which can accurately identify more than 99% of PWS and approximately 80% of AS [2, 6, 7]. *UBE3A* sequence analysis detects mutations in approximately a further 10% of patients with AS [6]. The multiplex-fluorescent-labeled STRs assay based linkage analysis was carried out to define the underlying genetic mechanisms. It can differentiate the molecular defects between typical deletion and UPD, exactly uniparental heterodisomy [2]. Establishing the mechanism will provide information on the possible clinical features, the prognosis and the recurrence risk [8]. Moreover, CMA was carried out to validate previous results and to detect the chromosomal breakpoints. It is an efficient and sensitive method for precisely detecting CNVs, and SNP-based CMA can directly identify isodisomic UPDs or identical by descents (IBDs) and associated mosaicisms. [9] The strategies adopted in the study for the analysis of PWS/AS demonstrated with satisfying performance. Yet the routine approaches did not include methylation-specific multiplex ligation dependent probe amplification (MS-MLPA) analysis, which can simultaneously detect copy number changes and DNA methylation defects within chromosome 15q11–13 region in a semi-quantitative manner [10], mainly out of the financial consideration. Since the costs of genetic testing for PWS/AS were not covered by the Medicare scheme in China, more cost-effective approaches were employed for the routine detection.

As to the study cohort, the majority of suspected individuals (3241 out of 3331) were neonates (≤28 days) presented with initial symptoms of PWS/AS, such as poor reflexes, sucking and swallowing difficulties. Given that the characteristic signs evolve with age and the initial symptoms overlap with other disorders, it could be difficult for the clinical diagnosis of PWS/AS in early infancy. As previous studies indicated [2, 5, 10, 11], the median age of diagnosis in PWS patients varied between 0.8 and 19.8 years, and in AS patients it was 0.9 to 6.2 years. In the present study, genetic screening was carried out in suspected individuals from neonatal intensive care units. The median age of diagnosis was 12 days in PWS patients and 22 days in AS patients. The prognosis of confirmed patients has improved significantly by early and continued therapies. Furthermore, by carefully analyzing the clinical manifestations of PWS/AS neonates in the present study and retrospectively viewing the special features of Asian patients in previous researches [2, 7, 10–13], a better understanding of clinical manifestations in Asian PWS/AS patients was learned. It would facilitate the differential diagnosis and early referral, and could result in early diagnosis and better management for PWS/AS patients.

Moreover, in this cohort, 83.3% of PWS were caused by paternal microdeletion, 16.7% by UPD(15)mat; while 85.7% of AS were caused by maternal microdeletion, 7.1% by UPD(15)pat, and 7.1% by *UBE3A* gene mutation. The distributin pattern of underlying meachanisms in this cohort was compared with those in other published cohorts. In western populations like United States and Germany, about 70–75% of PWS were caused by paternal microdeletion and 20–25% by UPD(15)mat; whereas in Asian regions like Japan, Korea and Taiwan of China, 80% of PWS were caused by paternal microdeletion and 15–20% by UPD(15)mat. The distributin of underlying meachanisms in our cohort of PWS patients was similar to the patterns in Asian PWS cohorts. Besides, in previous published cohorts, 70–75% of AS were caused by maternal microdeletion, 2–7% by UPD(15)pat, 3–5% by IC defect and about 5–10% by *UBE3A* mutation. The distributin of underlying meachanisms in our cohort of AS patients was similar to the patterns in published AS cohorts, only the proportion of AS caused by maternal microdeletion was comparetively higher than in other cohorts. Microdeletion type was the most common reported mechanism for AS. One of the explanation was chromosome 15q11–13 region harbored multiple low copy repeats (LCRs) that mediated recurrent homologous rearrangement like deletion, duplication and inversion which made this imprinted region as one of the most variable regions in the human genome. Another explanation for its higher proportion might due to ethnic backgorund. The cohort in our study was composed of Chinese Han population, and most of Southern China ancestries. Yet the difference might still be caused by bias in the distributin due to the limited size of the AS patient cohort. Genetic counseling was offered to families with affected individuals, concerning the nature of the diseases, genetic etiology of different molecular classes, interventions and prognosis of PWS/AS patients. Assessment of recurrence risk was carried out based on the genetic mechanism of the proband [4, 5].

## Conclusions

A practical set of molecular genetic testing has been adopted for the diagnosis of PWS/AS in the clinical practice of Guangdong Province, and demonstrated with comparatively satisfying performance. A better understanding of clinical manifestations and underlying meachanisms in Chinese PWS/AS patients was learned through this comparetively large cohort of 36 PWS patients and 14 AS patients. Identifying the disorders at early age, establishing the molecular mechanisms, carrying out treatment intervention and close monitoring can significantly improve the prognosis of PWS/AS patients.

## Abbreviations

AS: Angelman syndrome
CMA: Chromosomal microarray analysis
IBDs: Identical by descents
LCRs: Low copy repeats
MS-PCR: Methylation-specific PCR
PWS: Prader-Willi syndrome
STRs: Short tandem repeats
UPD: Uniparental disomy
VOUS: Variants of unknown significance
